# A novel system for micron‐scale analysis of energy deposition and response to low‐dose radiation

**DOI:** 10.1002/mp.70344

**Published:** 2026-02-27

**Authors:** Prarthana Pasricha, Connor McNairn, Iymad R. Mansour, Kirsty Milligan, Bryan R. Muir, Jeffrey L. Andrews, Edana Cassol, Vinita Chauhan, Sanjeena Subedi, Andrew Jirasek, Sangeeta Murugkar, Rowan M. Thomson

**Affiliations:** ^1^ Department of Physics Carleton University Ottawa Ontario Canada; ^2^ Radiation Medicine Program Princess Margaret Cancer Centre Toronto Ontario Canada; ^3^ Department of Radiation Oncology University of Toronto Toronto Ontario Canada; ^4^ Department of Physics University of British Columbia—Okanagan Campus Kelowna British Columbia Canada; ^5^ Metrology Research Centre National Research Council of Canada Ottawa Ontario Canada; ^6^ Department of Statistics University of British Columbia—Okanagan Campus Kelowna British Columbia Canada; ^7^ Department of Health Sciences Carleton University Ottawa Ontario Canada; ^8^ Consumer and Clinical Radiation Protection Bureau Health Canada Ottawa Ontario Canada; ^9^ Department of Statistics Carleton University Ottawa Ontario Canada

**Keywords:** low‐dose radiation, microdosimetry, Monte Carlo, optical density, Raman spectroscopy

## Abstract

**Background:**

Micrometer‐scale evaluation of energy deposition is important for radiation protection and therapy as well as for advancing knowledge of responses to radiation in materials and biological systems. Due to the stochastic nature of radiation interactions, there is significant variation in energy deposition in micrometer‐sized targets, especially at low doses. This variability underscores the need for a framework for microdosimetry, particularly in low‐dose scenarios.

**Purpose:**

The goal of this work is to develop a novel system for micron‐scale characterization of energy deposition and response to radiation that is applicable at low doses, using a combination of Monte Carlo (MC) simulations and experimental techniques.

**Methods:**

EBT3 radiochromic film samples are irradiated to absorbed doses of 0.003–0.5 Gy using the 6‐MV beam from a clinical linear accelerator. To quantify energy deposition, MC simulations of the experimental irradiations are conducted to evaluate specific energy deposited within micron‐scale voxels in the active layer of the film. To investigate the dose response of the film, the following two methods are employed: (i) flatbed scanner measurement of changes in optical density (OD) of the film, and (ii) Raman spectroscopy (RS) to measure response intensity across doses with micron‐scale resolution. Experimental film responses are compared to predictions from the microdosimetric one‐hit model.

**Results:**

Specific energy distributions obtained from MC simulations show large variation in energy deposition at low doses and within small targets; the “microdosimetric spread” (relative standard deviation) is significantly higher (>10 times) at 0.003 Gy than at 0.5 Gy, and is observed to decrease with increases in dose and target size. Both RS and OD measurements exhibit a near linear dose–response relationship, reflecting the film's sensitivity across micro‐ and macroscopic spatial scales. Overall, the OD and RS values determined using the one‐hit model with MC‐obtained specific energy distributions fit well to experimental measurements, with percentage differences up to 15 and 9.8%, respectively. An initial comparison of the relative standard deviation of RS and OD measurements (corrected for offset signal) shows qualitative agreement with the trends observed for MC‐determined microdosimetric spread.

**Conclusion:**

This study provides first results of a system that combines simulations with experimental techniques to investigate radiation response in micron‐scale targets, with a focus on low‐dose radiation exposure. The system shows promise in enabling future investigations of energy deposition within small volumes at low doses, where biological responses may be heterogeneous as some cells may receive high energy deposits and incur damage, while others may experience minimal or no deposition.

## 1 INTRODUCTION

Micrometer‐scale evaluation of energy deposition is critical for a wide range of applications, from emerging therapeutic approaches, such as microbeam therapy, nanoparticle‐enhanced treatments, and targeted radiation therapies, to radiation protection in contexts like diagnostic imaging, healthy tissue sparing, and occupational exposure. In addition to its practical relevance, assessment of energy deposition on micrometer scales also plays a key role in advancing our understanding of how materials and biological systems respond to radiation. Microdosimetry provides a theoretical framework for quantifying the stochastic nature of radiation interactions.^1^ It defines quantities to characterize energy deposition in microscopic targets, including the specific energy, z (energy imparted per unit mass in a volume of interest or target), and its distribution, f(z,D), within a population of targets when the absorbed dose is D. Specific energy is the stochastic analogue of absorbed dose, D, which is generally equal to the mean specific energy, $\bar{z}$. For small radiation targets and/or low doses, D no longer provides a reliable estimate of the energy deposited in targets—microdosimetric quantities such as z and f(z,D) must be considered.

Monte Carlo (MC) simulations offer the possibility of exploring dosimetry across length scales, with recent developments offering insights into energy deposition relevant for microdosimetry, all with the aim of better understanding the biological effects of radiation on living tissues. Villegas et al. used MC techniques to investigate microdosimetric “spread,” $\sigma_{z}/\bar{z}$, the standard deviation in specific energy relative to the mean, considering cubic voxels of side length 5–13 μm to approximate cell nuclei for 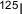, 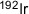, and 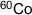 photon sources.^2^ Oliver and Thomson investigated energy deposition in subcellular targets using MC models of normal and cancerous human soft tissues, demonstrating that specific energy distributions depend on target size and dose magnitude for photon sources.^3^ Multiscale modeling for diagnostic breast imaging has shown the considerable variation in specific energy distributions in glandular cells, for example, with the traditional metric of mean glandular dose differing by factors of up to 10 compared to specific energies in glandular cells for mammography.^4^, ^5^, ^6^ Wang et al. used simple geometric models of a cell population as well as monolayer mesh‐type cell models to quantify specific energy distributions within cell nuclei^7^; furthermore, they used 10× Genomics single cell sequencing technology to investigate the heterogeneity of individual cell responses to low‐dose radiation in the same irradiated sample.

Experimental methods to assess energy deposition for microdosimetry continue to evolve. Tissue‐equivalent proportional counters have long been the gold standard; however, their large size, high voltage requirements, wall effect, and susceptibility to pile‐up effects limit their application.^8^, ^9^ Solid‐state detectors have emerged as an alternative, offering small physical sizes, high spatial resolution, and the ability to withstand high‐intensity beams without requiring gas supply or high voltage operation.^1^, ^10^ Silicon‐based microdosimeters leverage well‐established microfabrication techniques to create precisely defined sensitive volumes (SVs), enabling accurate measurements in complex radiation fields.^1^, ^10^, ^11^ The primary limitation of solid‐state microdosimeters is their relatively high threshold to detect the minimum imparted energy, due to a combination of detector and preamplifier noise.^1^ Silicon‐on‐insulator (SOI) devices feature 3D cylindrical SVs designed to mimic cellular dimensions, enhancing their applicability in radiobiological studies.^12^, ^13^, ^14^

Radiochromic film has been identified as a candidate for micron‐scale dosimetry.^15^, ^16^, ^17^ Gafchromic EBT3 film optical properties changes upon irradiation. EBT3 is comprised of a 28‐μm-thick active layer, sandwiched between two 125‐μm-thick polyester layers. The active layer consists of densely packed stick‐like monomer crystals of lithium pentacosa‐10,12‐diynoate (LiPCDA), a form of diacetylene.^18^ Upon irradiation, monomers polymerize, forming a linear long chained polymer (polyPCDA) with alternating double and triple carbon–carbon bonds in the backbone; polyPCDA concentration increases and film darkens with absorbed dose.^19^, ^20^, ^21^ Flatbed optical scanners are commonly employed for film readout by evaluating their optical density (OD) on spatial resolutions of 85–350 μm.^22^, ^23^ One‐hit detector theory has been successfully applied to predict the dose and energy response of EBT film,^24^, ^25^, ^26^ including in the context of microdosimetry.^27^

More recently, evaluation of film radiation response with spatial resolution on scales of a few microns has been demonstrated using Raman spectroscopy (RS) techniques.^15^, ^16^, ^17^ RS makes use of the inelastic scattering of light resulting from interactions with specific vibrational modes of chemical bonds in molecules; the quantity of radiation sensitive Raman active bonds in the polymer backbone increases with increasing radiation‐induced film polymerization.^28^ Recent work investigated the potential for achieving spatial resolution on the order of 1–2 μm for RS readout of film irradiated to doses of 0.2–2 Gy with 6‐MV photons.^29^ RS has also been used to probe cellular responses to radiation across doses relevant to radiation protection and therapy. For the former, researchers have demonstrated the potential for RS to assess response on subcellular (μm) levels and at low doses. For example, changes have been observed in Raman spectra for human lymphocytes irradiated to doses as low as 0.05 Gy,^30^ human lens epithelial cells and doses down to 0.01 Gy,^31^ and human keratinocyte cells and doses as low as 0.005 Gy.^32^ Considerable microdosimetric spread is predicted for these small targets and low doses, for example, 37% at 0.05 Gy for human lymphocytes, 175% at 0.01 Gy for human lens epithelial cells, and 57% at 0.005 Gy for human keratinocytes, however this has been overlooked in previous studies.^33^

The current work focuses on the development of a novel system to evaluate energy deposition on micron‐scales that is sensitive to low doses, using a combination of MC simulations and experimental techniques. EBT3 radiochromic film samples are irradiated with a 6‐MV linac beam to doses ranging from 0.003 to 0.5 Gy. To quantify energy deposition, MC simulations of the experimental irradiations are conducted to evaluate specific energy distributed within micron‐scale voxels in the active layer of the film. To investigate the dose response of the film, two methods are employed: (i) flatbed scanner film readout of OD (the standard technique for film readout^34^), and (ii) Raman spectroscopic measurement of intensity response with micron‐scale resolution. Experimental results are compared with predictions from the application of one‐hit detector theory using specific energy distributions obtained from MC simulations. The aim of this study is to explore the potential of a system that combines simulations with experimental techniques to assess energy deposition and response in a population micron‐scale targets.

## 2 MATERIALS AND METHODS

**FIGURE 1:**
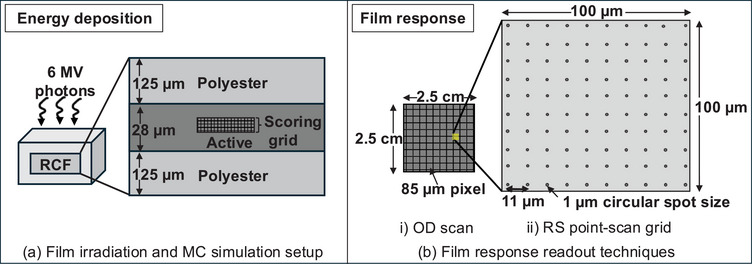
Schematic diagrams of the methods used to quantify (a) energy deposition using Monte Carlo (MC) simulations and (b) film response using (i) optical density (OD) and (ii) Raman spectroscopy (RS) measurements.

GAFchromic EBT3 film (Ashland Specialty Ingredients, NJ, USA)^19^ is used in this study, owing to its tissue equivalence, energy independent response, and high spatial resolution. Schematic diagrams providing an overview of the methods are shown in Figure 1. The film irradiation procedure is described in Section 2.1. MC simulations used to evaluate energy deposition within the film are described in Section 2.2, followed by a description of the two approaches used to analyze the film response in Section 2.3. Finally, the microdosimteric one‐hit detector theory and its application to predict the dose response of the film is discussed in Section 2.4.

### 2.1 EBT3 film irradiations

The EBT3 film samples of area $2.5\times 2.5\;{\text{cm}}^{2}$ are cut from a single sheet, and marked to ensure consistency in sample orientation. Irradiations of the film samples are performed using the Elekta Synergy clinical linear accelerator at the National Research Council of Canada, with the 6‐MV photon beam directed perpendicular to the plane of the film samples. For each irradiation, the film is placed at the center of a $30\times 30\;{\text{cm}}^{2}$ Virtual Water (Med‐Tec) phantom at a depth of 5 cm and with 5‐cm backscatter.

An extended source to surface distance (SSD) is utilized to enable delivery of low doses. The SSD is set to 215 cm, implemented with a vertical beam geometry by positioning the Virtual Water phantom on the floor. The collimation is set to $5\times 5\;{\text{cm}}^{2}$ to deliver a field size of approximately $10\times 10\;{\text{cm}}^{2}$ at the Virtual Water phantom surface for all doses.

The film samples are irradiated to the following doses: 0.003, 0.005, 0.007, 0.01, 0.02, 0.03, 0.04, 0.05, 0.07, 0.09, 0.1, 0.2, 0.3, 0.4, 0.5 Gy. To check for any drift in linac delivery, the delivered dose per monitor unit (MU), measured using a calibrated secondary standard reference chamber (type PTW30013) connected to an electrometer (Keithley 6517A), is measured both before and after irradiations. The uncertainty in dose delivery is reported to be 2.6% for doses greater than 0.2 Gy, increasing to 4.2% at 0.003 Gy, primarily due to increased dose monitor linearity error for low MU settings.^35^

### 2.2 Monte Carlo simulations

MC simulations of the experimental setup used for film irradiations are conducted using the EGSnrc^36^ application egs_brachy,^37^ from the EGSnrc_CLRP v2023a github egs_brachy branch with most recent commit 0219903. Benchmarking and verification that are relevant to the current work include tests on cell scale geometries.^3^, ^38^ The transport cutoff and production threshold for the kinetic energy of electrons and photons is 1 keV. The XCOM photon cross‐section^39^ and the NRC bremsstrahlung cross‐section databases^36^ are used. Rayleigh scattering, bound Compton scattering, spin effects, and electron impact ionization are turned on. The high‐resolution random number generator is used. Other transport parameters are set to their default values as specified in the EGSnrc MC code manual.^36^ Variance reduction techniques are not applied.

As in the experimental irradiations, the $2.5\times 2.5\;{\text{cm}}^{2}$ film sample is modeled at the center of a $30\times 30\times 10\;{\text{cm}}^{3}$ solid water phantom (see Figure 1). The mass elemental compositions used for the polyester and active layers of the EBT3 film are from Palmer et al.^40^ The 6‐MV photon spectrum from Sheikh‐Bagheri et al.^41^ is used for the radiation source. A parallel photon beam with $10\times 10\;{\text{cm}}^{2}$ cross‐section is oriented along the *Z*‐axis, and specific energy is scored in voxels within the film's active layer. Two sets of simulations are performed for all doses, differing only in the sizes and number of scoring voxels: (i) “vx‐sm”: scoring array of 1024×1024×4 voxels with dimensions $0.8862\times 0.8862\times 6\;\mu m^{3}$, corresponding to the sampling volume used in RS studies; (ii) “vx‐lg”: 1024×1024×2 array of voxels with dimensions $1.436\times 1.436\times 9.4\;\mu m^{3}$, representing the average size of monomer crystals in the active layer of the film.^20^ The number of voxels along the *Z*‐axis in both simulation sets is chosen to fit within the 28‐μm thickness of the active layer in the EBT3 film.

Absorbed doses (mean specific energy, $\bar{z}$) ranging from 0.003 to 0.5 Gy are achieved by varying the number of simulated histories. Simulations are run on the Narval and Beluga clusters (provided by the Digital Research Alliance of Canada), with approximately $5\times 10^{7}$ histories processed per hour per CPU, and each simulation requiring between 100 and 15 000 CPU hours (each dose represents a separate simulation). Statistical uncertainties on absorbed dose are estimated by replacing the array of scoring voxels by a single scoring volume spanning their collective volume and computing the uncertainty using history‐by‐history approach^42^: uncertainties thus calculated vary with dose and are less than <1%.

From the specific energies z recorded via MC simulation, the following quantities are calculated and compared across doses: mean ($\bar{z}$), relative standard deviation ($\sigma_{z}/\bar{z}$), normalized distribution (f(z,D); with $\int_{0}^{\infty }f\left(z,D\right)\;dz=1$), and the fraction of targets receiving no energy deposition ($f_{z=0}$).

### 2.3 Measurement of film response

#### 2.3.1 Optical density measurements

A flatbed scanner, Epson 10000XL, is used to read all film samples. To minimize the effects of lateral dependence artifacts caused due to the light scattering of the scanner lamp caused by particles in the film's active layer, a plastic template is fitted to the scanner to ensure that the film is positioned at a reproducible central location of the scan surface that can be considered uniform. The images are acquired in transmission mode and the orientation of the film is kept consistent. The irradiated film samples are scanned in the 48‐bit RGB mode (16 bits per color), with a resolution of 300 dpi and 85‐μm pixel size. Each film sample is scanned three times. Additionally, pixel averaging is applied during post‐processing: groups of three adjacent pixels are averaged, effectively reducing the spatial resolution to 100 dpi and further suppressing noise. For every channel, the scanner response values are converted to net OD using an in‐house Python routine, and an average value of net OD for each sample is obtained. For the dose range in which we are interested, only the net OD values from the red channel are considered to obtain the sensitometric curve (dose vs. OD). The OD is defined by the following: $\text{OD}=\log_{10}\left(\frac{I_{0}}{I}\right)$, where $I_{0}$ and I are the film readings (i.e., pixel values of the transmission scan) for control and irradiated film sample, respectively.

#### 2.3.2 Raman spectra measurement

The Raman micro‐spectroscopy technique used for this study follows an approach similar to that of McNairn et al.,^29^ and key aspects of the technique are outlined in this section. A custom‐built Raman microscope features a 785‐nm single‐mode fiber‐coupled laser with a power output of 60 mW. To minimize laser‐induced polymerization, a neutral OD filter is added, resulting in a laser power of 4 mW at the sample. Using a 60× 1.1 numerical aperture water immersion objective (Olympus, Canada), the laser is focused to a lateral spot of diameter 1 μm with a depth of focus of 6 μm. A quarter‐wave plate is added to circularly polarize the light, eliminating polarization dependence within the measured spectra. The back‐scattered Raman signal is filtered with a dichroic mirror and two long‐pass edge filters, collected through a 100‐μm diameter optical fiber, and delivered to a Raman spectrometer with a 100‐μm slit and a 600‐line ${\mathrm{mm}}^{{-}^{1}}$ grating. Spectra are measured in the 614–2313 ${\mathrm{cm}}^{-1}$ range with a spectral resolution of 4 ${\mathrm{cm}}^{-1}$. The data collection time for each spectrum is set to 1 s, with five spectra acquired at each pixel, resulting in a total of 500 spectra per sample. After allowing sufficient time (>1 day) for the polymerization in the films to stabilize, the irradiated films are secured on a custom‐made stainless‐steel slide to ensure flatness and consistent orientation. The *Z*‐axis position is adjusted by focusing on the back‐reflected laser spot, and the stage was vertically translated to maximize the Raman signal at 1445 ${\mathrm{cm}}^{-1}$ from the polymer in the active layer. Raman spectra are acquired using an automated *X*–*Y* translation of the film sample through the laser focus over a 100 × 100 μm 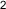 region of interest (ROI), with data collected from a 10 × 10 grid pattern, resulting in 500 spectra per sample. Raman measurements are completed in a single day, with each dataset repeated three times. To correct for decreased spectrometer sensitivity at higher Raman shifts, Raman spectra of a fluorescence standard are measured twice during the testing period, and standard samples of polystyrene and silicon are measured before each set of film measurements.

The preprocessing of the Raman spectra data follows established techniques from previous studies.^17^, ^29^ A modified cosmic‐ray algorithm removed artifacts caused by cosmic ray interactions. Five spectra per pixel are averaged to create a representative spectrum for each pixel. Background subtraction is performed using the sensitive nonlinear iterative peak‐clipping (SNIP) algorithm and the NIST correction curve. For calibration curves, spectra are vector normalized to minimize variance in signal due to slight differences in measurement conditions between datasets and then normalized to the area under a dose‐independent Raman peak centered at 2260 ${\mathrm{cm}}^{-1}$ and of width 14 ${\mathrm{cm}}^{-1}$.^29^

### 2.4 Film response prediction with one‐hit detector theory

The one‐hit detector model refers to types of detectors showing a linear response at low doses and saturating exponentially for higher doses. The one‐hit model provides the theoretical framework to express the number of centers that are hit, and as the name suggests, relies on the assumption that a single quantum of ionizing radiation is sufficient to induce an effect, in this case, the formation of a polymer in an active center in the film.^26^, ^27^ The LiPCDA crystals within the active layer of the EBT3 film function as the SVs that respond to radiation. Zaider's microdosimetric one‐hit detector model^43^ defines the dose response of a one‐hit detector with a large number of similar SVs using $R_{\text{theory}}$, the fraction of affected SVs in the detector with the following equation:

(1) $$R_{\text{theory}}\left(D\right)=1-\int_{0}^{\infty }\exp\left(-\alpha \cdot z\right)\cdot f\left(z;D\right)\;dz$$

where α is the saturation parameter, which is the probability that a unit increment of specific energy results in a hit, and f(z;D) is the MC‐computed microdosimetric distribution in z at dose D.

Moslehi et al.^27^ proposed a linear relationship between OD and the fraction of affected SVs, $R_{\text{theory}}\left(D\right)$. We modify Equation (1) by introducing a scaling factor, $m_{\text{OD}}$, and a constant representing the offset signal, $C_{\text{OD}}$, to predict the response of the film in terms of OD:

(2) $$R_{\text{OD}}\left(D\right)=m_{\text{OD}}\left(1-\int_{0}^{\infty }\exp\left(-\alpha_{\text{OD}}\cdot z\right)\cdot f\left(z;D\right)\;dz\right)+C_{\text{OD}}.$$

Similarly, as the Raman intensity signal from the film also increases with the number of affected SVs, we assume an analogous relation for the response as measured by RS, $R_{\text{RS}}\left(D\right)$, allowing for a different scaling factor ($m_{\text{RS}}$), saturation parameter ($\alpha_{\text{RS}}$), and offset signal ($C_{\text{RS}}$). Experimental measurements of film response using the flatbed scanner are fit to Equation (2) using MC‐generated specific energy distributions in voxels of size corresponding to the average size of the monomer crystals in the active layer of the film (vx‐lg). Least squares fitting is performed with the Levenberg Marquardt algorithm to obtain $m_{\text{OD}}$, $\alpha_{\text{OD}}$, and $C_{\text{OD}}$, for the dose range 0.003–0.5 Gy; the process is analogous for RS measured response.

## 3 RESULTS

### 3.1 Specific energy distributions

Specific energy distributions obtained from MC simulations considering two different target sizes and varying doses are presented in this section. These distributions, the microdosimetric spread $\sigma_{z}/\bar{z}$, and the fraction of targets that receive no energy deposition ($f_{z=0}$) exhibit significant dependence on both dose and target size. The stochastic nature of radiation interactions is particularly pronounced at lower doses and for smaller target size, where fewer energy deposition events occur in each voxel. This results in a larger number of voxels with no energy deposition and a broader microdosimetric spread. At higher doses, the number of energy deposition events per voxel increases, reducing stochastic variations in specific energy deposition. Similarly, for a given dose, larger targets experience more deposition events, leading to a more uniform distribution of specific energy. In both cases, averaging over a greater number of events reduces statistical variation.

**FIGURE 2:**
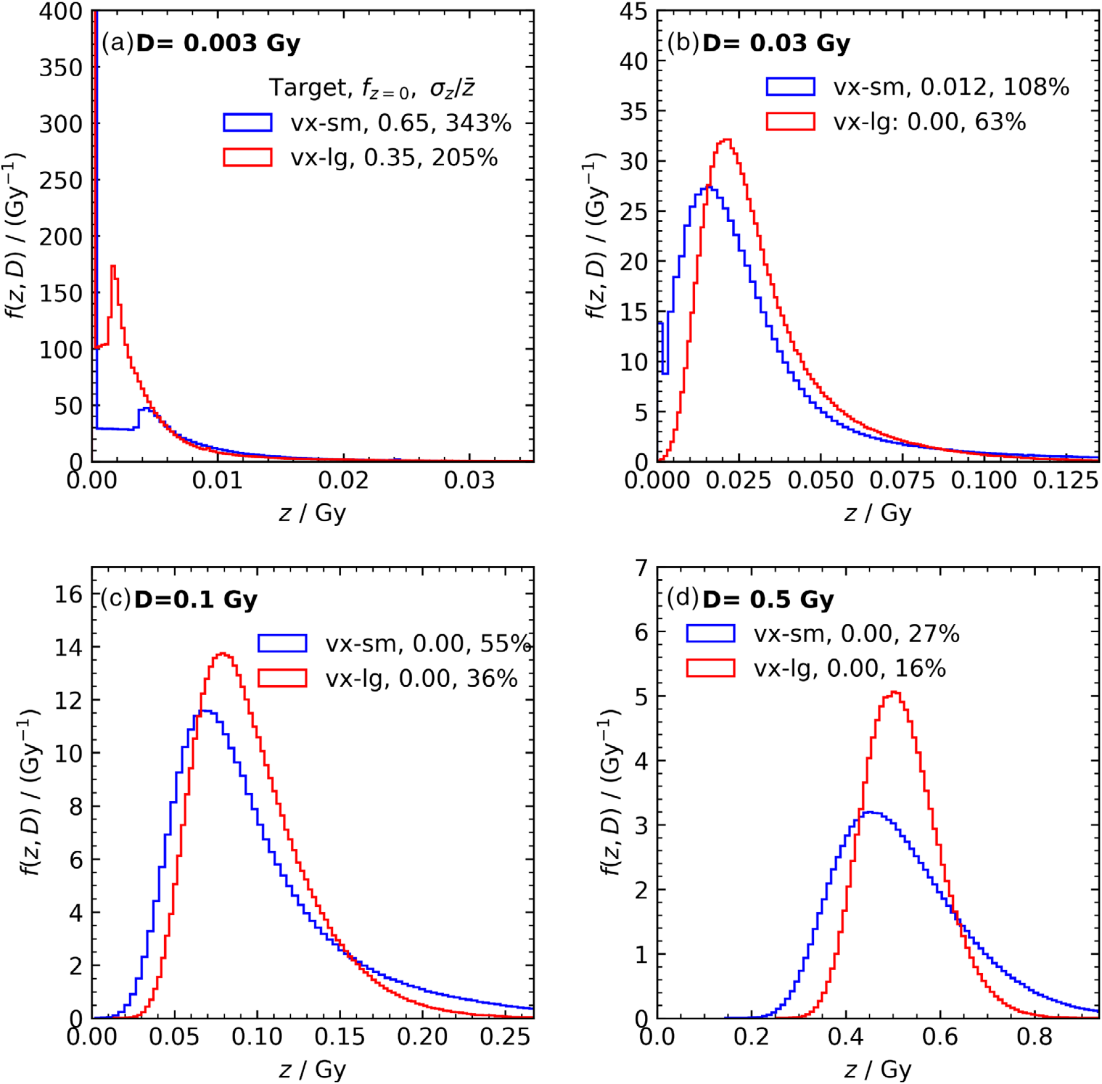
Specific energy distributions for small voxels (vx‐sm: $0.8862\times 0.8862\times 6\;\mu m^{3}$; corresponding to the Raman sampling volume) and larger voxels (vx‐lg: $1.436\times 1.436\times 9.4\;\mu m^{3}$; corresponding to the average size of crystals in the film's active layer) for a subset of doses. The fraction of targets receiving no energy deposition and the relative standard deviation of the specific energy are given in the legend.

Figure 2 illustrates the specific energy distributions for two different target sizes, for four representative absorbed doses. In general, at the lower doses, many voxels receive no energy and the distributions are skewed; with increasing dose, the distributions become narrower and less skewed. At the relatively low dose of 0.003 Gy, the fraction of voxels with zero energy deposition ($f_{z=0}$) is 0.65 and 0.35, while $\sigma_{z}/\bar{z}$ is 343 and 205% for small (vx‐sm: $0.8862\times 0.8862\times 6\;\mu m^{3}$) and large (vx‐lg: $1.436\times 1.436\times 9.4\;\mu m^{3}$) voxel sizes, respectively. The shape of the specific energy distributions at these low doses is affected by the distribution of chord lengths for electrons crossing the voxels^1^; the peak observed near 0.005 Gy for vx‐sm (0.002 Gy for vx‐lg) corresponds to the energy deposited by an electron (from a Compton interaction at the mean photon energy of the spectrum) crossing the mean chord length of the voxel. This is consistent with, for example, Massera et al.^5^ who observed a peak corresponding to energy deposition from a single photoelectric interaction in MC‐determined specific energy histograms for glandular cells in digital mammography. As the dose increases to 0.5 Gy, all voxels receive some energy ($f_{z=0}=0$), though variation in specific energy deposition remains, with $\sigma_{z}/\bar{z}$ values of 27 and 15% for small and large voxel sizes, respectively. Results for the full range of doses considered in this study are summarized in Table 1.

**TABLE 1:** Summary of MC simulation results: dose and corresponding statistical uncertainty (%unc.), fraction of targets receiving no energy ($f_{z=0}$), and relative standard deviation of specific energy ($\frac{\sigma_{z}}{\bar{z}}\left(\%\right)$) for two different voxel volumes.

(a) vx‐sm				(b) vx‐lg			
Dose		f(z,D)		Dose		f(z,D)	
Gy	%unc.	$f_{z=0}$	$\frac{\sigma_{z}}{\bar{z}}\left(\%\right)$	Gy	%unc.	$f_{z=0}$	$\frac{\sigma_{z}}{\bar{z}}\left(\%\right)$
0.003	0.84	0.65	343	0.003	0.53	0.35	205
0.006	0.61	0.40	237	0.006	0.43	0.099	138
0.009	0.52	0.24	189	0.009	0.35	0.074	131
0.011	0.42	0.16	167	0.011	0.29	0.033	114
0.02	0.30	0.053	132	0.02	0.21	0.0006	78
0.03	0.26	0.012	108	0.03	0.17	0	63
0.04	0.21	0	95	0.04	0.15	0	55
0.05	0.20	0	88	0.05	0.13	0	46
0.07	0.17	0	74	0.07	0.11	0	43
0.09	0.15	0	63	0.09	0.10	0	39
0.1	0.14	0	55	0.1	0.091	0	36
0.2	0.10	0	41	0.2	0.080	0	25
0.3	0.078	0	35	0.3	0.067	0	20
0.4	0.061	0	33	0.4	0.054	0	17
0.5	0.058	0	27	0.5	0.0043	0	15

**FIGURE 3:**
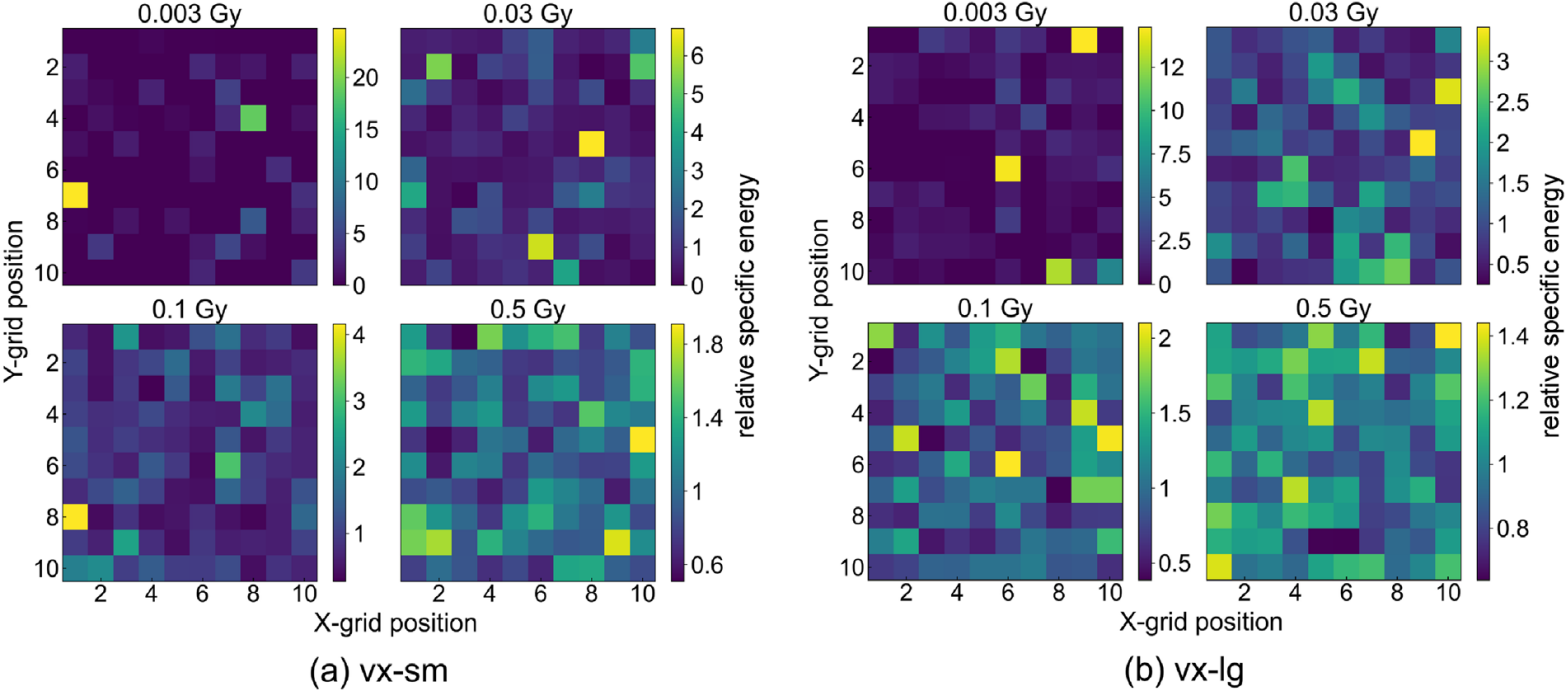
Two‐dimensional maps of specific energy deposited in 10 × 10 sampled voxel grid, shown for the four representative doses. Each map is normalized to the average specific energy in the given ROI.

To assess homogeneity of dose deposition, two‐dimensional spatial distributions are analyzed using an ROI defined by an array of 100×100 voxels from the specific energy distribution dataset for a subset of doses, considering both voxel sizes. Figure 3 presents two‐dimensional specific energy maps on a 10×10 voxel grid sampled from the central 100×100 voxels in a manner representative of the RS sampling grid. Each map depicts the specific energy deposited in the sampled voxels, normalized to the nominal specific energy within the corresponding ROI. A dose‐dependent pattern is observed, with high spatial heterogeneity at lower doses (0.003 and 0.03 Gy), characterized by discrete regions of elevated specific energy deposition. In contrast, at higher doses (0.1 and 0.5 Gy), the distribution becomes more homogeneous, suggesting a reduction in stochastic fluctuations in energy deposition with increasing dose.

### 3.2 Film response

#### 3.2.1 Optical density

**FIGURE 4:**
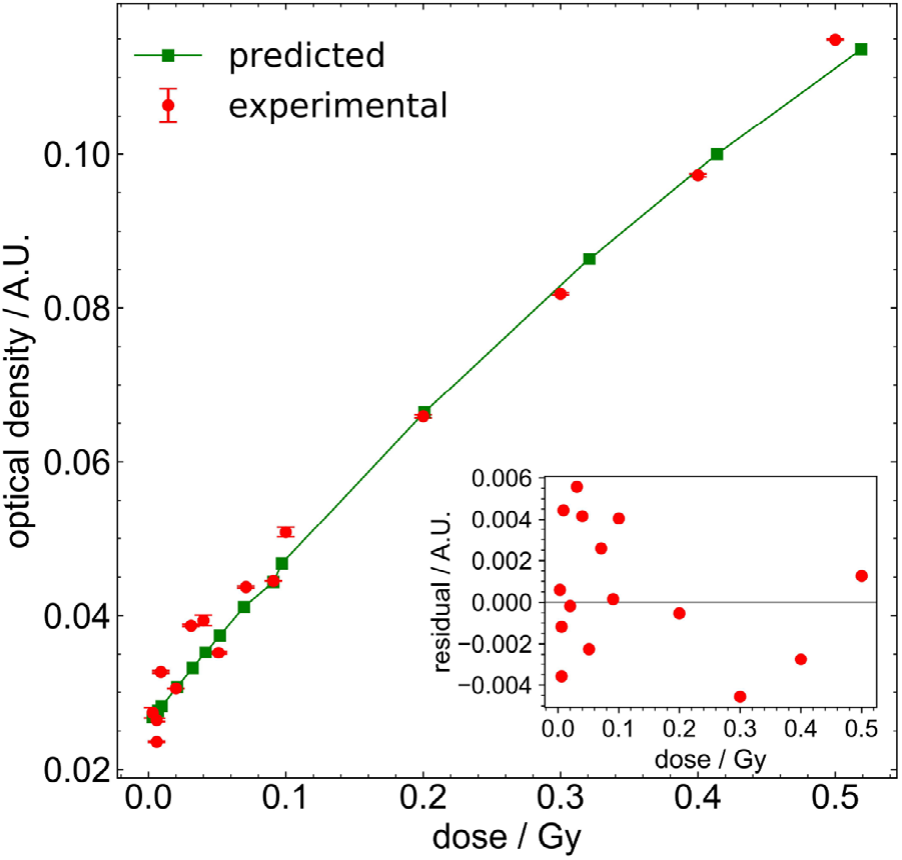
Experimental net optical density of EBT3 film as a function of dose, compared with the predicted response $R_{\text{OD}}\left(D\right)$ (Equation 2) derived from the microdosimetric one‐hit model. The solid line is fit to the predicted data, with residuals between experimental data and model predictions shown in the subplot.

Figure 4 shows that the OD increases with dose over the range considered of 0.003–0.5 Gy. The nonzero OD at 0 Gy indicates an offset signal from polymerization, unrelated to ionizing radiation.^29^ Considering two‐dimensional OD data, the standard deviation of the OD in the central 100 × 100 pixels is approximately constant across the doses considered; thus, the standard deviation expressed as a percentage of the OD decreases with dose, from 76% at 0.003 Gy to 7.4% at 0.5 Gy.

Predicted OD values from the one‐hit model, evaluated using Equation (2) and the experimental results, along with the residuals are presented in Figure 4 for the full dose range; fit parameters are found to be $m_{\text{OD}}=0.19$, $\alpha_{\text{OD}}=1.2$ ${\mathrm{Gy}}^{-1}$ and $C_{\text{OD}}=0.026$. Overall, the OD values determined using the one‐hit model with MC‐obtained specific energy distributions fit well to experimental measurements, with percentage differences ranging from 0.05 to 15%.

#### 3.2.2 Raman spectra

**FIGURE 5:**
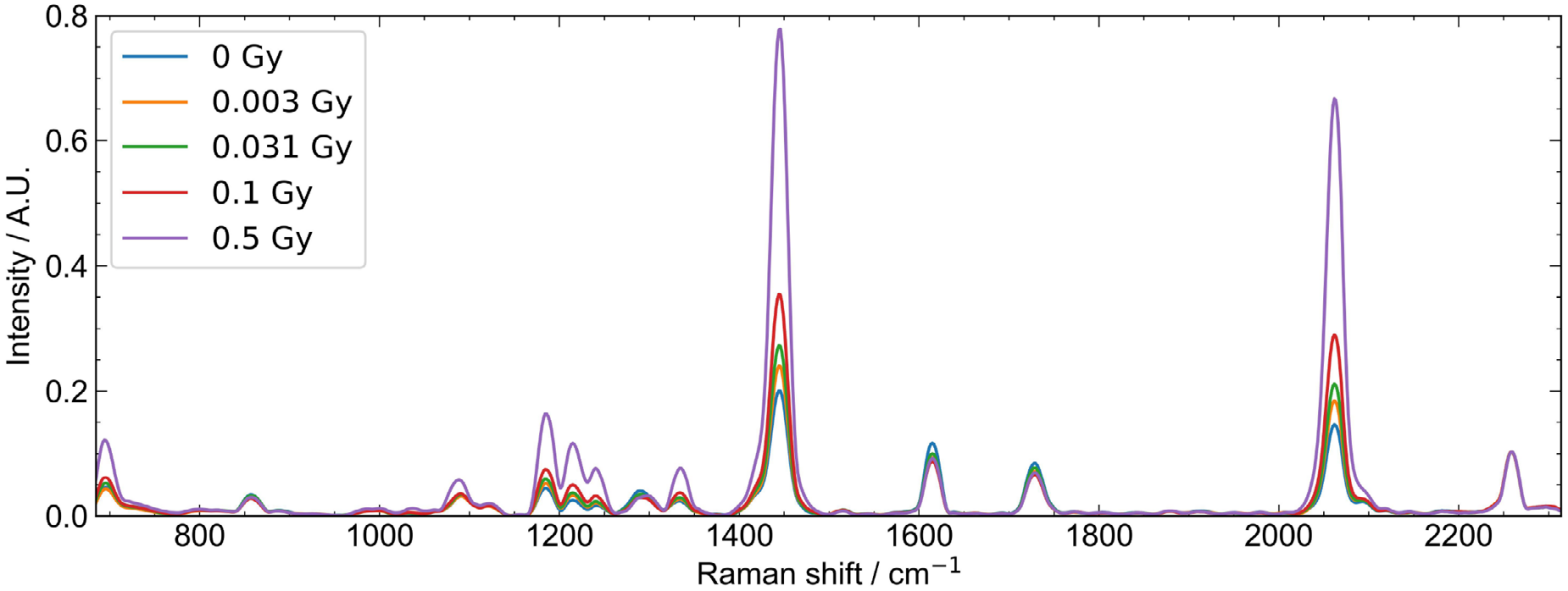
Raman spectra (after data preprocessing) are shown for four representative doses and a control sample (0 Gy). Each spectrum shown is the mean (*n* = 500) of the Raman spectral response of films measured with a 60× objective over the $100\times 100\;\mu m^{2}$.

**FIGURE 6:**
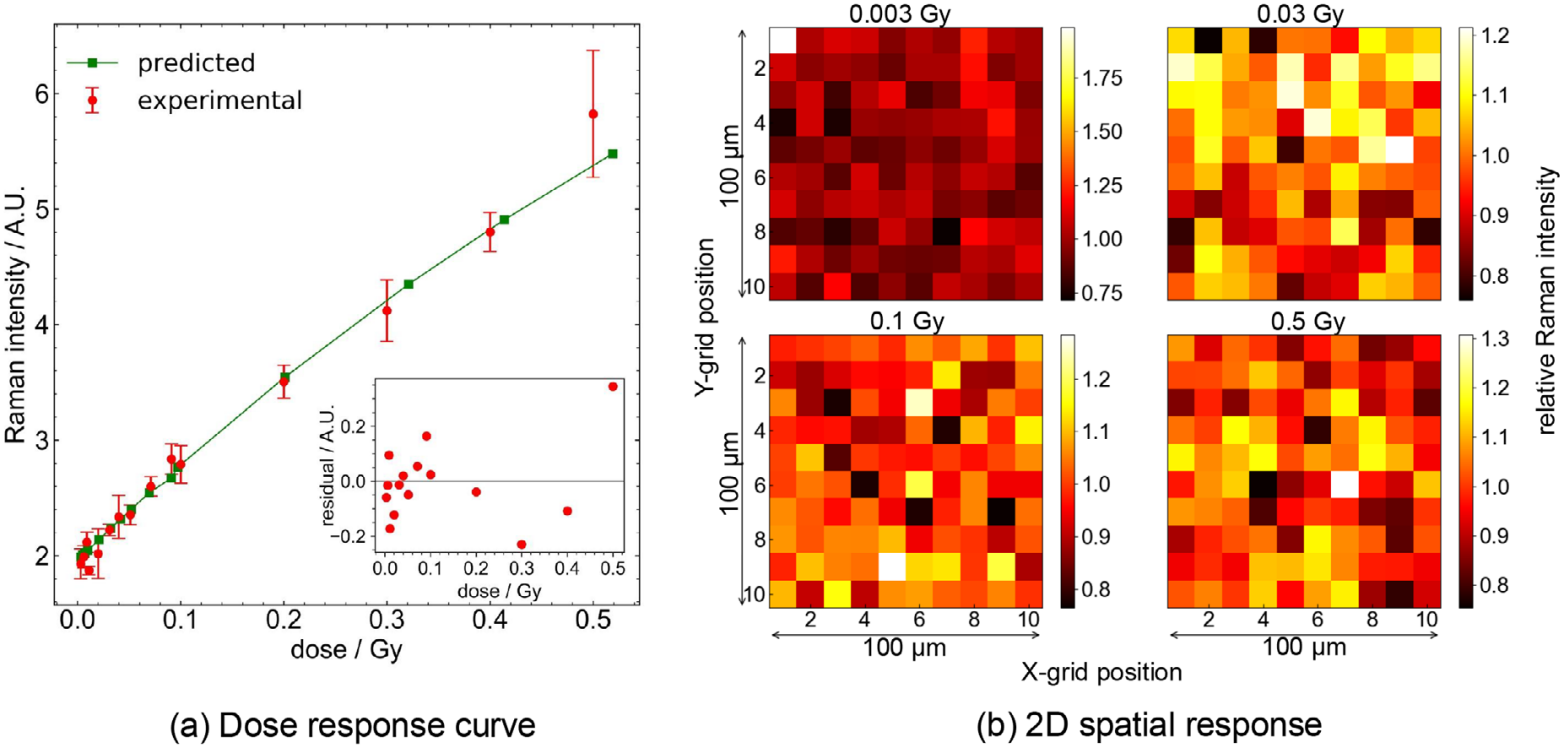
(a) Dose–response curve obtained from preprocessed spectra(withdata averaged over three datasets), shown for both experimental measurements and model predictions based on the microdosimetric one‐hit theory. (b) Two‐dimensional maps of the intensity of the Raman peaks at 1445 ${\mathrm{cm}}^{-1}$ normalized to the monomer peak at 2260 ${\mathrm{cm}}^{-1}$, displayed over 10×10 grids spanning $100\times 100\;\mu m^{2}$, for four doses.

Figure 5 illustrates the average of 500 preprocessed Raman spectra acquired from a $100\times 100\;\mu m^{2}$ ROI in a 10×10 grid for doses 0.003, 0.03, 0.1, and 0.5 Gy and the control (0 Gy) sample. These spectra reflect a partially polymerized state, exhibiting signals from both monomer and polymer. The key Raman peaks are identified as follows: 696 ${\mathrm{cm}}^{-1}$ (δ(CCC) vibrations), 1086 ${\mathrm{cm}}^{-1}$ (ν(C−C) vibrations), 1445 ${\mathrm{cm}}^{-1}$ (ν(C=C) vibrations), and 2060 ${\mathrm{cm}}^{-1}$ (ν(C≡C) vibrations) within the film's active layer. The peak at 2260 ${\mathrm{cm}}^{-1}$ corresponds to the ν(C≡C) mode in the monomer crystals. The intensity of the Raman signal at 2260 ${\mathrm{cm}}^{-1}$ is found to be dose‐independent and served as an internal standard for normalization. Post‐normalization to the 2260 ${\mathrm{cm}}^{-1}$ peak, the mean and standard deviation of the Raman spectral intensity are determined for each pixel across the ROI. The averaged Raman response at 1445 ${\mathrm{cm}}^{-1}$, shown in Figure 6a, indicates a linear dose response within the 0.003–0.5‐Gy range, with a relative standard deviation ranging up to 5.5% across the three datasets. Note that the relative standard deviation across ROI, based on pixel intensity variations, ranges from 8.9 to 17% and does not appear to depend on dose. Figure 6b shows 2D maps from the ROI for a subset of doses. Considerable variation in the relative Raman intensity of individual pixels is seen compared to the nominal intensity of the ROI.

For the predicted RS response (from one‐hit detector theory), the fitting parameters are found to be $m_{\text{RS}}=8.6$, $\alpha_{\text{RS}}=1.02$ ${\mathrm{Gy}}^{-1}$, and $C_{\text{RS}\;}=1.96$. The RS values determined using the one‐hit model fit well to experimental measurements, with percentage differences ranging from 0.15 to 9.8%.

## 4 DISCUSSION

This study presents the first results of a novel system enabling micron‐scale analysis of energy deposition and radiation response at low doses ranging from 0.003 to 0.5 Gy. This is the first investigation (to our knowledge) that characterizes the macro‐ to microscopic response of EBT3 film at low dose levels using both OD and RS measurements. Results are compared with MC‐simulated specific energy distributions and agree well with predictions obtained from the application of one‐hit theory.

Analysis of specific energy distributions obtained from MC simulations reveals significant variations in energy deposition in relation to target size and dose. Microdosimetric spread is pronounced for the small voxels at lower doses, where the stochastic nature of radiation results in a higher fraction of voxels with zero‐energy deposition and a skewed specific energy distribution. Larger target sizes and higher doses reduce microdosimetric spread and enhance energy deposition averaging. These results are qualitatively consistent with findings from Oliver and Thomson.^33^ This means that in biological systems exposed to low doses, energy deposition events would be infrequent and randomly distributed, leading to significant fluctuations in the energy absorbed by individual cells. This variability can result in heterogeneous biological responses, where some cells receive higher energy deposits and may experience damage, while others receive minimal or no energy deposition. Such heterogeneity is important in understanding radiation effects, as it suggests that even low‐dose exposures can lead to varied cellular outcomes, potentially influencing risk assessments and therapeutic strategies.

For the dose range considered in this study, both RS and flatbed OD measures exhibit linear dose–response trends, even though they are originating from different underlying mechanisms. While OD is a quantitative measure of the absorbance of the film (logarithmic ratio between the light incident and the light transmitted through the film), RS probes the vibrational modes of radiation‐sensitive chemical bonds in the active layer of the film, providing molecular specificity. Nonetheless, the combination of the two techniques can provide complementary information about changes in the film at macroscopic and microscopic levels. An obvious limitation of the flatbed scanner is its limited spatial resolution and its applicability being restricted to films. On the other hand, RS shows tremendous promise in measurements of cell response,^29^ in addition to its use in conjunction with film for microdosimetry.

**FIGURE 7:**
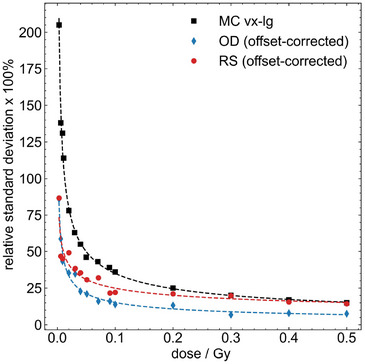
Preliminary comparison of relative standard deviation as a function of dose for MC results, and for RS and OD intensities after offset correction (0‐Gy signal subtraction). The dashed lines represent fitted power‐law trends and are shown here to guide the eye.

Initial analysis of the RS measurements of film response do not show the same trends as seen in specific energy distributions from MC simulations when comparing the relative standard deviation in intensity across regions of interest. The presence of an intrinsic offset signal at 0 Gy, arising from initial polymerization of the film obscures the subtle, radiation‐induced changes observed in Raman intensity, particularly at low doses. Consequently, there are no “zero‐energy” voxels analogous to those present in the MC simulations. This interplay masks expected trends in the specific energy distribution with dose. As an initial attempt to address this, we implement a signal offset correction by subtracting the mean Raman intensity of an unirradiated (i.e., 0 Gy) sample from that of each irradiated sample on a pixel‐by‐pixel basis. This effectively corrects the film to zero signal at 0 Gy. After applying this “offset correction,” the trends in the relative standard deviation of the RS intensity more closely resemble the microdosimetric spread determined through MC simulation. However, the magnitude of the relative standard deviation remains lower than expected, highlighting the need for improved signal offset correction methods and more robust modeling of the film's inherent inhomogeneity. A similar correction is performed for the OD signal and the results are shown in Figure 7.

Del Moral et al. studied four theoretical models to characterize the sensitometric response of EBT film, ultimately determining that the one‐hit theory most accurately describes the film's behavior in the dose range of 0–2 Gy.^24^ In the present work, microdosimetric one‐hit model predictions show good fit with experimental measurements, with differences from experimental data up to 15 and 9.8% for OD and RS measurements, respectively. These discrepancies may be partly due to the use of uniform voxels—identical in size and orientation—which does not accurately represent the actual distribution of crystals in the film's active layer. In a spectroscopic study of EBT3 by Callens et al., they found that two distinct conformations of the PDA polymer (blue and red phase) with different absorption properties are formed.^28^ They conclude that the polymer phases have different dose‐dependent reaction kinetics. Hence, in MC simulations, consideration of different crystal sizes within the film rather than having a fixed size could reduce these differences but is yet to be tested at low‐dose range.

Future work will aim to address the limitations identified in this study to enable a direct mapping between MC‐simulated energy deposition and Raman‐based measurements of micron‐scale response. The presence of an intrinsic offset signal in the unirradiated film and the nonuniform dispersion of monomer crystals in active layer of the RCF pose a limitation for using EBT3 films in experimental microdosimetry. To overcome this, improved signal offset correction methods and statistical approaches capable of decoupling film‐related heterogeneity from that of the radiation‐induced energy deposition are warranted. An alternative solution may be to transition to newer film, EBT4, which has undergone process improvements to its active fluid, reportedly leading to enhanced signal‐to‐noise ratio and improved response characteristics.^44^ From the simulation perspective, a more realistic approach would involve replacing the assumption of uniformly distributed SVs with a model incorporating randomly distributed and size‐varying SVs, better reflecting the true structure of the active layer in EBT3 films. Additionally, it would be valuable to assess the feasibility and performance of this system under lower‐energy radiation exposures, such as kilovoltage x‐ray beams, which are commonly used in certain preclinical and diagnostic applications.

## 5 CONCLUSION

This study provides first results of a system that combines simulations with experimental techniques to investigate radiation response in cell‐scale targets, with a specific focus on low‐dose (0–0.5 Gy) radiation exposure. MC studies of energy deposition reveal substantial microdosimetric spread, that is, variation in energy deposition, at low doses and small target volumes. Considering biological systems, this means that at these levels, energy deposition events are infrequent and randomly distributed, leading to significant fluctuations in the energy absorbed by individual cells. Both RS and OD measurements of film response exhibit a near linear dose–response relationship, reflecting the film's sensitivity across micro‐ and macroscopic spatial scales. The trends in the relative standard deviation of the RS measurement (corrected for offset signal) qualitatively resemble those observed in MC simulations. However, the magnitude of the relative standard deviation remains lower than expected, highlighting the need for improved signal offset correction methods and more robust modeling of the film's inherent inhomogeneity. Microdosimetric one‐hit model predictions using MC‐determined specific energy distributions fit well to experimental measurements. Our ongoing efforts focus on developing a statistical modeling framework to isolate and quantify different sources of variation in the RS‐based film response, facilitating direct comparisons with MC‐derived energy deposition data. The outcomes of this work can contribute towards establishing a system for microdosimetry applicable to investigate radiation response in biologically relevant cellular systems.

## CONFLICT OF INTEREST STATEMENT

The authors declare no conflicts of interest.

## References

[1] Braby LA , Conte V , Dingfelder M , et al. ICRU report 98, stochastic nature of radiation interactions: microdosimetry. J ICRU. 2023;23(1):1‐168. doi:10.1177/14736691231211380

[2] Villegas F , Tilly N , Ahnesjö A . Microdosimetric spread for cell‐sized targets exposed ${\mathrm{to}}^{60}$ Co, Ir ${\mathrm{and}}^{125}$ I sources. Radiat Prot Dosim. 2015;166(1‐4):365‐368. doi:10.1093/rpd/ncv200 25911409

[3] Oliver PAK , Thomson RM . Investigating energy deposition within cell populations using Monte Carlo simulations. Phys Med Biol. 2018;63(15):155018. doi:10.1088/1361-6560/aacf7b 29947613

[4] Oliver PAK , Thomson RM . Investigating energy deposition in glandular tissues for mammography using multiscale Monte Carlo simulations. Med Phys. 2019;46(3):1426‐1436. doi:10.1002/mp.13372 30657190

[5] Massera RT , Tomal A , Thomson RM . Multiscale Monte Carlo simulations for dosimetry in x‐ray breast imaging: part I ‐ macroscopic scales. Med Phys. 2024;51(2):1105‐1116. doi:10.1002/mp.16910 38156766

[6] Massera RT , Tomal A , Thomson RM . Multiscale Monte Carlo simulations for dosimetry in x‐ray breast imaging: part II ‐ microscopic scales. Med Phys. 2024;51(2):1117‐1126. doi:10.1002/mp.16912 38146824

[7] Wang Y , Gao J , Tang B , et al. A comparative study on the dose‐effect of low‐dose radiation based on microdosimetric analysis and single‐cell sequencing technology. Sci Rep. 2024;14(1):11524. doi:10.1038/s41598-024-62501-5 38773212 PMC11109114

[8] Rademacher SE , Borak TB , Zeitlin C , Heilbronn L , Miller J . Wall effects observed in tissue‐equivalent proportional counters from 1.05 GeV/nucleon iron‐56 particles. Radiat Res. 1998;149(4):387‐395. doi:10.2307/3579702 9525504

[9] Booz J , Braby L , Coyne J , et al. 1. Introduction. Rep Int Commission Radiat Units and Meas . 1983;os‐19(1):1‐3. doi:10.1093/jicru_os19.1.1

[10] Bradley P , Rosenfeld A , Zaider M . Solid state microdosimetry. Nucl Instrum Methods Phys Res Sect B: Beam Interact Mater Atoms. 2001;184(1‐2):135‐157. doi:10.1016/S0168-583X(01)00715-7 11863030

[11] Kemmer J , Burger P , Henck R , Heijne E . Performance and applications of passivated ion‐implanted silicon detectors. IEEE Trans Nucl Sci. 1982;29(1):733‐737. doi:10.1109/TNS.1982.4335947

[12] Rosenfeld AB . Novel detectors for silicon based microdosimetry, their concepts and applications. Nucl Instrum Methods Phys Res Sect A: Accel, Spectrom, Detect Assoc Equip. 2016;809:156‐170. doi:10.1016/j.nima.2015.08.059

[13] Tran LT , Chartier L , Bolst D , et al. In‐field and out‐of‐file application in 12C ion therapy using fully 3D silicon microdosimeters. Radiat Meas. 2018;115:55‐59. doi:10.1016/j.radmeas.2018.06.015

[14] Tran LT , Bolst D , James B , et al. Silicon 3D microdosimeters for advanced quality assurance in particle therapy. Appl Sci. 2021;12(1):328. doi:10.3390/app12010328

[15] Mirza JA , Park H , Park SY , Ye SJ . Use of radiochromic film as a high‐spatial resolution dosimeter by Raman spectroscopy: Raman spectroscopy dosimetry using radiochromic films. Med Phys. 2016;43(8 pt 1):4520‐4528. doi:10.1118/1.4955119 27487869

[16] Mirza JA , Hernández Millares R , Kim GI , Park S , Lee J , Ye S . Characterization of radiochromic films as a micrometer‐resolution dosimeter by confocal Raman spectroscopy. Med Phys. 2019;46(11):5238‐5248. doi:10.1002/mp.13778 31442302

[17] Mcnairn C , Mansour I , Muir B , Thomson RM , Murugkar S . High spatial resolution dosimetry with uncertainty analysis using Raman micro‐spectroscopy readout of radiochromic films. Med Phys. 2021;48(8):4610‐4620. doi:10.1002/mp.15000 34042192

[18] Rink A , Lewis DF , Varma S , Vitkin IA , Jaffray DA . Temperature and hydration effects on absorbance spectra and radiation sensitivity of a radiochromic medium. Med Phys. 2008;35(10):4545‐4555. doi:10.1118/1.2975483 18975701 PMC2736758

[19] Ashland Specialty Ingredients. 2024. https://www.ashland.com/industries/medical/radiotherapy‐films

[20] Callens MB , Crijns W , Depuydt T , et al. Modeling the dose dependence of the vis‐absorption spectrum of EBT3 GafChromic films. Med Phys. 2017;44(6):2532‐2543. doi:10.1002/mp.12246 28370086

[21] Lewis D , Micke A , Yu X , Chan MF . An efficient protocol for radiochromic film dosimetry combining calibration and measurement in a single scan. Med Phys. 2012;39(10):6339‐6350. doi:10.1118/1.4754797 23039670 PMC9381144

[22] Borca VC , Pasquino M , Russo G , et al. Dosimetric characterization and use of GAFCHROMIC EBT3 film for IMRT dose verification. J Appl Clin Med Phys. 2013;14(2):158‐171. doi:10.1120/jacmp.v14i2.4111 PMC5714357 23470940

[23] Massillon‐JL G , Chiu‐Tsao ST , Domingo‐Munoz I , Chan MF . Energy dependence of the new GafChromic EBT3 film: dose response curves for 50 kV, 6 and 15 mV x‐ray beams. Int J Med Phys, Clin Eng Radiat Oncol. 2012;1(02):60‐65. doi:10.4236/ijmpcero.2012.12008 PMC13387173 28517140

[24] Del Moral F , Vázquez JA , Ferrero JJ , et al. From the limits of the classical model of sensitometric curves to a realistic model based on the percolation theory for GafChromic EBT films. Med Phys. 2009;36(9):4015‐4026. doi:10.1118/1.3187226 19810474

[25] Martín‐Viera Cueto JA , Parra Osorio V , Moreno Sáiz C , Navarro Guirado F , Casado Villalón FJ , Galán Montenegro P . A universal dose–response curve for radiochromic films. Med Phys. 2015;42(1):221‐231. doi:10.1118/1.4903301 25563262

[26] Hammer CG , Rosen BS , Fagerstrom JM , Culberson WS , DeWerd LA . Experimental investigation of GafChromic^®^ EBT3 intrinsic energy dependence with kilovoltage x rays, , and . Med Phys. 2018;45(1):448‐459. doi:10.1002/mp.12682 29159807

[27] Moslehi A , Ansari M , Monadi S . Microdosimetric modeling of the sensitometric curve of GafChromic films in the photon fields. Physica Medica. 2020;69:170‐175. doi:10.1016/j.ejmp.2019.12.020 31918369

[28] Callens M , Crijns W , Simons V , et al. A spectroscopic study of the chromatic properties of GafChromic EBT3 films. Med Phys. 2016;43(3):1156‐1166. doi:10.1118/1.4941312 26936701

[29] McNairn C , Pasricha P , Milligan K , et al. Exploring the potential of Raman micro‐spectroscopy of radiochromic films for experimental microdosimetry. Med Phys. 2025;52(7):e17900. doi:10.1002/mp.17900 40665569 PMC12264321

[30] Maguire A , Vegacarrascal I , White L , et al. Analyses of ionizing radiation effects *in vitro* in peripheral blood lymphocytes with raman spectroscopy. Radiat Res. 2015;183(4):407‐416. doi:10.1667/RR13891.1 25844945

[31] Allen CH , Kumar A , Qutob S , Nyiri B , Chauhan V , Murugkar S . Raman micro‐spectroscopy analysis of human lens epithelial cells exposed to a low‐dose‐range of ionizing radiation. Phys Med Biol. 2018;63(2):025002. doi:10.1088/1361-6560/aaa176 29235993

[32] Meade AD , Howe O , Unterreiner V , Sockalingum GD , Byrne HJ , Lyng FM . Vibrational spectroscopy in sensing radiobiological effects: analyses of targeted and non‐targeted effects in human keratinocytes. Faraday Discuss. 2016;187:213‐234. doi:10.1039/C5FD00208G 27043923

[33] Oliver PAK , Thomson RM . Microdosimetric considerations for radiation response studies using Raman spectroscopy. Med Phys. 2018;45(10):4734‐4743. doi:10.1002/mp.13145 30141185

[34] Niroomand‐Rad A , Chiu‐Tsao S , Grams MP , et al. Report of AAPM Task Group 235 radiochromic film dosimetry: an update to TG‐55. Med Phys. 2020;47(12):5986‐6025. doi:10.1002/mp.14497 32990328

[35] Nithya L , Raj NN , Rathinamuthu S . Analyzing the characteristics of 6 MV photon beam at low monitor unit settings. J Med Phys. 2016;41(1):34‐37. doi:10.4103/0971-6203.177285 27051168 PMC4795415

[36] Kawrakow I , Mainegra‐Hing E , Rogers DWO , Tessier F , Walters BRB . The EGSnrc Code System: Monte Carlo Simulation of Electron and Photon Transport . Technical report. Ionizing Radiation Standards, National Research Council Canada; 2025.

[37] Chamberland MJP , Taylor REP , Rogers DWO , Thomson RM . Egs_brachy: a versatile and fast Monte Carlo code for brachytherapy. Phys Med Biol. 2016;61(23):8214‐8231. doi:10.1088/0031-9155/61/23/8214 27804922

[38] Martinov MP , Thomson RM . Technical note: taking EGSnrc to new lows: development of egs++ lattice geometry and testing with microscopic geometries. Med Phys. 2020;47(7):3225–3232. doi:10.1002/mp.14172 32277472

[39] Berger MJ , Hubbell JH , Seltzer SM , et al. XCOM: Photon Cross Sections Database. NIST Standard Reference Database 8 (XGAM). 2010.

[40] Palmer AL , Dimitriadis A , Nisbet A , Clark CH . Evaluation of Gafchromic EBT‐XD film, with comparison to EBT3 film, and application in high dose radiotherapy verification. Phys Med Biol. 2015;60(22):8741‐8752. doi:10.1088/0031-9155/60/22/8741 26512917

[41] Sheikh‐Bagheri D , Rogers DWO . Monte Carlo calculation of nine megavoltage photon beam spectra using the BEAM code. Med Phys. 2002;29(3):391‐402. doi:10.1118/1.1445413 11930914

[42] Walters BRB , Kawrakow I , Rogers DWO . History by history statistical estimators in the BEAM code system. Med Phys. 2002;29(12):2745‐2752. doi:10.1118/1.1517611 12512706

[43] Zaider M . Microdosimetry and Katz's track structure theory: I. One‐hit detectors. Radiat Res. 1990;124(1):S16‐S22. doi:10.2307/3577672 2236505

[44] Palmer AL , Nash D , Polak W , Wilby S . Evaluation of a new radiochromic film dosimeter, Gafchomic EBT4, for VMAT, SABR and HDR treatment delivery verification. Phys Med Biol. 2023;68(17):175003. doi:10.1088/1361-6560/aceb48 37499683

